# Multicenter phase II trial (SWOG S1609, cohort 51) of ipilimumab and nivolumab in metastatic or unresectable angiosarcoma: a substudy of dual anti-CTLA-4 and anti-PD-1 blockade in rare tumors (DART)

**DOI:** 10.1136/jitc-2021-002990

**Published:** 2021-08-02

**Authors:** Michael J Wagner, Megan Othus, Sandip P Patel, Chris Ryan, Ashish Sangal, Benjamin Powers, G Thomas Budd, Adrienne I Victor, Chung-Tsen Hsueh, Rashmi Chugh, Suresh Nair, Kirsten M Leu, Mark Agulnik, Elad Sharon, Edward Mayerson, Melissa Plets, Charles Blanke, Howard Streicher, Young Kwang Chae, Razelle Kurzrock

**Affiliations:** 1 Clinical Research Division, Fred Hutchinson Cancer Research Center, Seattle, Washington, USA; 2 Medical Oncology, University of Washington, Seattle, Washington, USA; 3 SWOG Statistical and Data Management Center/Fred Hutchinson Cancer Research Center, Seattle, Washington, USA; 4 Department of Medicine, UCSD Moores Cancer Center, La Jolla, California, USA; 5 Department of Medicine, OHSU, Portland, Oregon, USA; 6 Western Regional Medical Center, Goodyear, Arizona, USA; 7 Department of Internal Medicine, University of Kansas Medical Center, Kansas City, Kansas, USA; 8 Hematology and Medical Oncology, Cleveland Clinic, Cleveland, Ohio, USA; 9 Department of Medicine, University of Rochester, Rochester, New York, USA; 10 Loma Linda University, Loma Linda, California, USA; 11 Department of Medicine, University of Michigan, Ann Arbor, Michigan, USA; 12 Lehigh Valley Health Network, Allentown, Pennsylvania, USA; 13 Nebraska Methodist Health System, Omaha, Nebraska, USA; 14 Medical Oncology & Therapeutics Research, City of Hope Comprehensive Cancer Center, Duarte, California, USA; 15 Department of Medicine, Northwestern University, Chicago, Illinois, USA; 16 Cancer Therapy Evaluation Program, National Cancer Institute, Bethesda, Maryland, USA; 17 SWOG, Portland, Oregon, USA

**Keywords:** Sarcoma, Clinical Trials, Phase II as Topic, Drug Therapy, Combination, Immunotherapy

## Abstract

**Purpose:**

Angiosarcoma is a rare aggressive endothelial cell cancer with high mortality. Isolated reports suggest immune checkpoint inhibition efficacy in angiosarcoma, but no prospective studies have been published. We report results for angiosarcoma treated with ipilimumab and nivolumab as a cohort of an ongoing rare cancer study.

**Methods:**

This is a prospective, open-label, multicenter phase II clinical trial of ipilimumab (1 mg/kg intravenously every 6 weeks) plus nivolumab (240 mg intravenously every 2 weeks) for metastatic or unresectable angiosarcoma. Primary endpoint was objective response rate (ORR) per RECIST 1.1. Secondary endpoints include progression-free (PFS) and overall survival, and toxicity. A two-stage design was used.

**Results:**

Overall, there were 16 evaluable patients. Median age was 68 years (range, 25–81); median number of prior lines of therapy, 2. Nine patients had cutaneous and seven non-cutaneous primary tumors. ORR was 25% (4/16). Sixty per cent of patients (3/5) with primary cutaneous scalp or face tumors attained a confirmed response. Six-month PFS was 38%. Altogether, 75% of patients experienced an adverse event (AE) (at least possibly related to drug) (25% grade 3–4 AE); 68.8%, an immune-related AE (irAE) (2 (12.5%), grade 3 or 4 irAEs (alanine aminotransferase/aspartate aminotransferase increase and diarrhea)). There were no grade 5 toxicities. One of seven patients in whom tumor mutation burden (TMB) was assessed showed a high TMB (24 mutations/mb); that patient achieved a partial response (PR). Two of three patients with PDL1 immunohistochemistry assessed had high PDL1 expression; one achieved a PR.

**Conclusion:**

The combination of ipilimumab and nivolumab demonstrated an ORR of 25% in angiosarcoma, with three of five patients with cutaneous tumors of the scalp or face responding. Ipilimumab and nivolumab warrant further investigation in angiosarcoma.

**Trial registration number:**

NCT02834013.

## Introduction

Angiosarcomas are aggressive cancers that are difficult to treat and have high mortality. As they are ultrarare tumors, with only approximately 400 new cases per year in the USA, therapy options for patients with metastatic disease are limited.^1 2^ Angiosarcomas are often responsive to chemotherapy, with response rates to taxanes ranging from 18% to 89% in several studies.^3–6^ However, these responses are not durable; the median progression-free survival (PFS) ranges from 4 to 9.5 months.^3–6^ Five-year survival for all patients with angiosarcoma, including those who present with localized disease, is only 30%–40%.^7^

A subset of angiosarcomas are characterized by high tumor mutation burden (TMB), suggesting that they may respond to immune checkpoint inhibitors.^8–11^ Initial immune characterization of soft tissue sarcomas included three angiosarcomas, and showed that all three had infiltration of PD-L1-expressing lymphocytes and macrophages.^12^ Larger series have reported varying levels of PD-L1 expression, with Tumor Proportion Scores (TPS) ranging from 14% to 66%.^13 14^ A series of seven angiosarcomas showed one with high PD-L1 expression.^13^ Two larger series have more recently been published, one reporting that 6 of 24 angiosarcoma samples (66%) of different origins, including bone, skin, breast, soft tissue, and visceral primary tumors, had at least some membrane expression of PD-L1 without a clear correlation with site of origin.^15^ Although angiosarcomas are characterized by dysregulated angiogenesis, there is no correlation between expression of PD-L1 or tumor infiltrating lymphocytes and vascular endothelial growth factor related gene expression.^14^

Published case reports and small series have demonstrated encouraging initial clinical responses to immune checkpoint inhibition (ICI) in patients with angiosarcoma. For instance, one of three patients with angiosarcomas enrolled on a phase II study of checkpoint inhibitors in sarcoma had an objective response.^16^ Two separate cases are also reported of patients with durable complete responses (CR) in angiosarcomas of the scalp^17^ and nose.^18^ A larger retrospective series of seven patients suggested a response rate of over 50%.^19^ These anecdotal cases suggest that checkpoint inhibition is active in a subset of angiosarcomas, and prompted the addition of an angiosarcoma cohort to DART (S1609) (Dual anti-CTLA-4 and anti-PD-1 Blockade in Rare Tumors), a prospective phase II study conducted through the National Cancer Institute (NCI)-supported SWOG Cancer Research Network’s Early Therapeutics and Rare Cancers Committee.

## Patients and methods

### Patients and procedures

DART is a multicenter (>900 sites), open-label, multiple cohort study of nivolumab and ipilimumab for rare malignancies. The Cancer Therapy Evaluation Program of the NCI provided study medication under a NCI Cooperative Research and Development Agreement agreement with Bristol-Myers Squibb. Angiosarcomas represented one of the 53 cohorts on the DART trial. The clinical protocol was conducted in accord with the Declaration of Helsinki. The study design and eligibility criteria for DART were as previously reported.^20^ All participants provided written informed consent authorized by the enrolling center’s internal review board.

Eligible patients in this cohort must have had a confirmed diagnosis of angiosarcoma with disease evaluable as per (Response Evaluation Criteria in Solid Tumours) RECIST 1.1^21^ Cutaneous only disease was allowed provided that it could be measured and followed with color photography. Patients were required to be at least 18 years of age and have adequate end organ function, including hematologic, renal, hepatic, adrenal, and thyroid function.

Patients received nivolumab 240 mg every 2 weeks and ipilimumab 1 mg/kg every 6 weeks (both intravenously). Disease assessments were performed at baseline and thereafter at weeks 8, 16, 24, and then every 12 weeks until tumor progression.

### Endpoints

The primary endpoint of the study was objective response rate (ORR) (confirmed complete and partial response (CR and PR, respectively)) as assessed by RECIST 1.1 measurement.^21^ Given historical data, we set the null hypothesis to be an ORR of 5%. The regimen was considered of interest for further study if the true ORR is 30% or higher (alternative hypothesis). Subset analyses within the cohort were not prespecified.

This cohort used a two-stage design. If >1 response was observed in the first six eligible and evaluable patients, accrual to the second stage to a total of 16 patients would be opened. Two or more responses out of 16 patients were considered evidence that the treatment regimen merits further investigation, pending other data including adverse events (AEs) also appear satisfactory. This design has 87% power with a one-sided alpha of 13%. If the true ORR is 5% or less, the probability of stopping accrual after the first stage was 74%; if the true ORR is 30% or greater, the probability of stopping accrual after the first stage was 12%.

PFS was measured from the initiation of study treatment to the first date of progression by RECIST 1.1 or death for any reason, with participants last known to be alive without progression censored at the date of last communication. Overall survival (OS) was evaluated from the date of clinical trial registration to the date of death, with patients last known to be alive censored at the date of last contact. PFS and OS estimates were calculated utilizing the Kaplan-Meier method^22^; they were compared using log-rank tests. CIs for the primary ORR analysis accounted for the two-stage design. All analyses were performed using R version 3.4.3.

## Results

### Patient characteristics

Overall, 18 patients from 11 National Clinical Trial Network institutions were registered between July 31, 2019 and March 19, 2020, with 16 patients meeting eligibility criteria and receiving protocol therapy (table 1). Two patients were excluded who were ineligible; one patient had baseline lab values outside protocol parameters and another for whom no clinical information was available after initial registration.

**Table 1:** Patient Summary

Characteristic	Summary (Median (min, max) or N (%))
Age (years)	68 (25, 81)
Gender	
Female	6 (38)
Male	10 (62)
Performance status	
0	7 (44)
1	9 (56)
Ethnicity	
Hispanic	2 (12)
Not Hispanic	14 (88)
Race	
White	13 (81)
Black	2 (12)
Unknown race	1 (6)
Primary site	
Breast	4 (25)
Extremity	2 (12)
Face/scalp	5 (31)
Heart	1 (6)
Liver	2 (12)
Spleen	1 (6)
Stomach	1 (6)
Cutaneous primary	
No	7 (44)
Yes	9 (56)
Radiation associated	
No	13 (81)
Yes	3 (19)
No prior therapies	2 (0, 5)

Of the 16 patients who were enrolled and eligible, median age was 68 years (range 25–81 years). Five patients had cutaneous primary tumor of the skin on the face or scalp and four had primary cutaneous tumors of other sites including two with radiation-associated cutaneous tumors on the breast. Six patients had visceral or non-cutaneous extremity tumors. One had a primary breast tumor. Median number of prior systemic therapies was 2 (online supplemental table 1). Molecular characterization done as part of routine medical care was available for eight patients (table 2).

10.1136/jitc-2021-002990.supp2 Supplementary data

**Table 2:** Genomic alterations in patients (N=8) whose tumors were assessed by clinical-grade NGS

Primary tumor site	Assay	TMB (mut/mb)	PDL1 status (Antibody)	NGS findings (characterized alterations; no VUSs)	Best response
Right atrium	Tissue NGS (FoundationOne Heme Panel, 405 genes) genes)	3	Not done	BRAF G469R, MLL2 Q52* and W2818*	PD
Scalp	Tissue NGS (FoundationOne Heme Panel, 406 genes)	8	TPS 50% (Ventana SP263 antibody)	*HRAS* and *HGF* amplification, *ATRX* splice site mutation, *TP53* A159V mutation	Died prior to first response assessment
Breast- XRT	Guardant 360 liquid biopsy NGS (74 genes)	Not done	Not done	*PEAR1-NTRK1* Fusion, *ATM* R337C, *TP53* T140fs, *MYC* amplification	PR
Breast	Tissue NGS (FoundationOne Heme Panel, 406 genes)	0	Not done	*PIK3CA P471L,* *HRAS G13D,* ASXL Q623fs*8, PRDM1 G585fs*48	PD
Skin of face	Tissue NGS (Tempus 1714 genes)	8.4	TPS 30% (22C3 antibody)	*CDKN2A* copy number loss, *POT1* p.Y122_E128delins*(LOF), *SPEN* p.R653* (LOF) *CDKN2B* copy number loss	PR
Scalp	Tissue NGS (local institutional panel, 170 genes)	24	Not done	*KIT* amplification, *TP53* A347V and E286K	PR
Spleen	Tissue NGS (Local Institutional Panel, 523 genes)	5	Not done	*ATM* R337H, *NOTCH1* c.2882delC:p.Thr961ArgfsTer218	SD (6+ months ongoing)
Skin of arm	Tissue NGS (FoundationOne Heme Panel, 406 genes)	5	TPS 0% (Ventana SP263 antibody)	*BRCA1* N1355fs*10, *CDKN2A/B* loss, *NOTCH1* V1575L	PD

LOF, loss of function; NGS, next generation sequencing; PD, progressive disease; PR, partial response; SD, stable disease; TMB, tumor mutation burden; TPS, Tumor Proportion Score; VUS, variant of uncertain significance; XRT, radiation therapy.

### Toxicities

Treatment-related AEs are summarized in table 3. Overall, 75% of patients experienced an AE, and 25% experienced a grade 3–4 AE. There were no drug-related deaths. In two patients (12.5%), toxicity led to drug discontinuation. One patient discontinued therapy due to grade 3 liver toxicity during cycle 3, and another discontinued for grade 3 lower limb muscle weakness after the first cycle. 2 patients discontinued ipilimumab alone but continued on nivolumab: one due to drug-induced hepatitis after three cycles and one due to grade 3 diarrhea after five cycles. The most common AEs each occurred in 18.8%: alanine aminotransferase (ALT) increase, anemia, aspartate aminotransferase (AST) increase, diarrhea, fatigue, hypothyroidism, pneumonitis, pruritus, and rash. Altogether, 68.8% of participants experienced an immune-related AE (irAE), and 2 (12.5%) developed grade 3 or 4 irAEs. The most common irAE occurred in 18.8% of patients each: ALT increase, AST increase, diarrhea, hypothyroidism, pneumonitis, pruritus, and rash. Grade 3–4 irAEs included ALT and AST increase, and diarrhea. No patient deaths were attributed to study drug.

**Table 3:** Treatment-related adverse events (N=16 patients)

	Any grade	Grade 3–4
Any	12 (75.0%)	4 (25.0%)
Serious	3 (18.8%)	2 (12.5%)
Led to discontinuation	2 (12.5%)	2 (12.5%)
Lead to death	0 (0.0%)	0 (0.0%)
>5% of Patients		
Alanine aminotransferase increased	3 (18.8%)	1 (6.3%)
Anemia	3 (18.8%)	1 (6.3%)
Aspartate aminotransferase increased	3 (18.8%)	1 (6.3%)
Diarrhea	3 (18.8%)	1 (6.3%)
Fatigue	3 (18.8%)	0 (0%)
Hypothyroidism	3 (18.8%)	0 (0%)
Pneumonitis	3 (18.8%)	0 (0%)
Pruritus	3 (18.8%)	0 (0%)
Rash maculo-papular	3 (18.8%)	0 (0%)
Alkaline phosphatase increased	2 (12.5%)	1 (6.3%)
Hypokalemia	2 (12.5%)	1 (6.3%)
Neutrophil count decreased	2 (12.5%)	1 (6.3%)
Fever	2 (12.5%)	0 (0%)
Hyponatremia	2 (12.5%)	0 (0%)
Infusion-related reaction	2 (12.5%)	0 (0%)
Insomnia	2 (12.5%)	0 (0%)
Lipase increased	2 (12.5%)	0 (0%)
Lymphocyte count decreased	2 (12.5%)	0 (0%)
Nausea	2 (12.5%)	0 (0%)
Vomiting	2 (12.5%)	0 (0%)
Hepatobiliary disorders—other, specify: drug-induced hepatitis	1 (6.3%)	1 (6.3%)
Infections and infestations—other, specify: drug-induced hepatitis	1 (6.3%)	1 (6.3%)
Muscle weakness lower limb	1 (6.3%)	1 (6.3%)
Pneumothorax	1 (6.3%)	1 (6.3%)
Anorexia	1 (6.3%)	0 (0%)
Arthralgia	1 (6.3%)	0 (0%)
Back pain	1 (6.3%)	0 (0%)
Bone pain	1 (6.3%)	0 (0%)
Dry mouth	1 (6.3%)	0 (0%)
Dry skin	1 (6.3%)	0 (0%)
Dysgeusia	1 (6.3%)	0 (0%)
Dyspnea	1 (6.3%)	0 (0%)
Endocrine disorders—other, specify: ACTH increased	1 (6.3%)	0 (0%)
Gastrointestinal pain	1 (6.3%)	0 (0%)
Hepatobiliary disorders—other, specify: immune-mediated hepatitis	1 (6.3%)	0 (0%)
Hyperglycemia	1 (6.3%)	0 (0%)
Hyperthyroidism	1 (6.3%)	0 (0%)
Hypocalcemia	1 (6.3%)	0 (0%)
Neuralgia	1 (6.3%)	0 (0%)
Pain in extremity	1 (6.3%)	0 (0%)
Platelet count decreased	1 (6.3%)	0 (0%)
Pleural effusion	1 (6.3%)	0 (0%)
Rash acneiform	1 (6.3%)	0 (0%)
Serum amylase increased	1 (6.3%)	0 (0%)
Weight loss	1 (6.3%)	0 (0%)
Immune-mediated	11 (68.8%)	2 (12.5%)
Alanine aminotransferase increased	3 (18.8%)	1 (6.3%)
Aspartate aminotransferase increased	3 (18.8%)	1 (6.3%)
Diarrhea	3 (18.8%)	1 (6.3%)
Hypothyroidism	3 (18.8%)	0 (0%)
Pneumonitis	3 (18.8%)	0 (0%)
Pruritus	3 (18.8%)	0 (0%)
Rash maculo papular	3 (18.8%)	0 (0%)
Infusion-related reaction	2 (12.5%)	0 (0%)
Lipase increased	2 (12.5%)	0 (0%)
Arthralgia	1 (6.3%)	0 (0%)
Hyperthyroidism	1 (6.3%)	0 (0%)
Serum amylase increased	1 (6.3%)	0 (0%)

### Genomic alterations and PDL1 expression

Eight of 16 patients had had NGS performed (table 2). All eight patients had >2 deleterious genomic alterations, and no two patients had the same set of genomic aberrations. One patient had a fusion involving *NTRK1*, one patient had an atypical *BRAF* mutation and one patient had a *BRCA1* alteration. One of the seven patients whose TMB was assessed had a high TMB. Of the three patients with programmed death-ligand 1 (PDL1) immunohistochemistry available, PDL1 TPS was 0%, 30% and 50%.

### Outcomes

**Figure 1:**
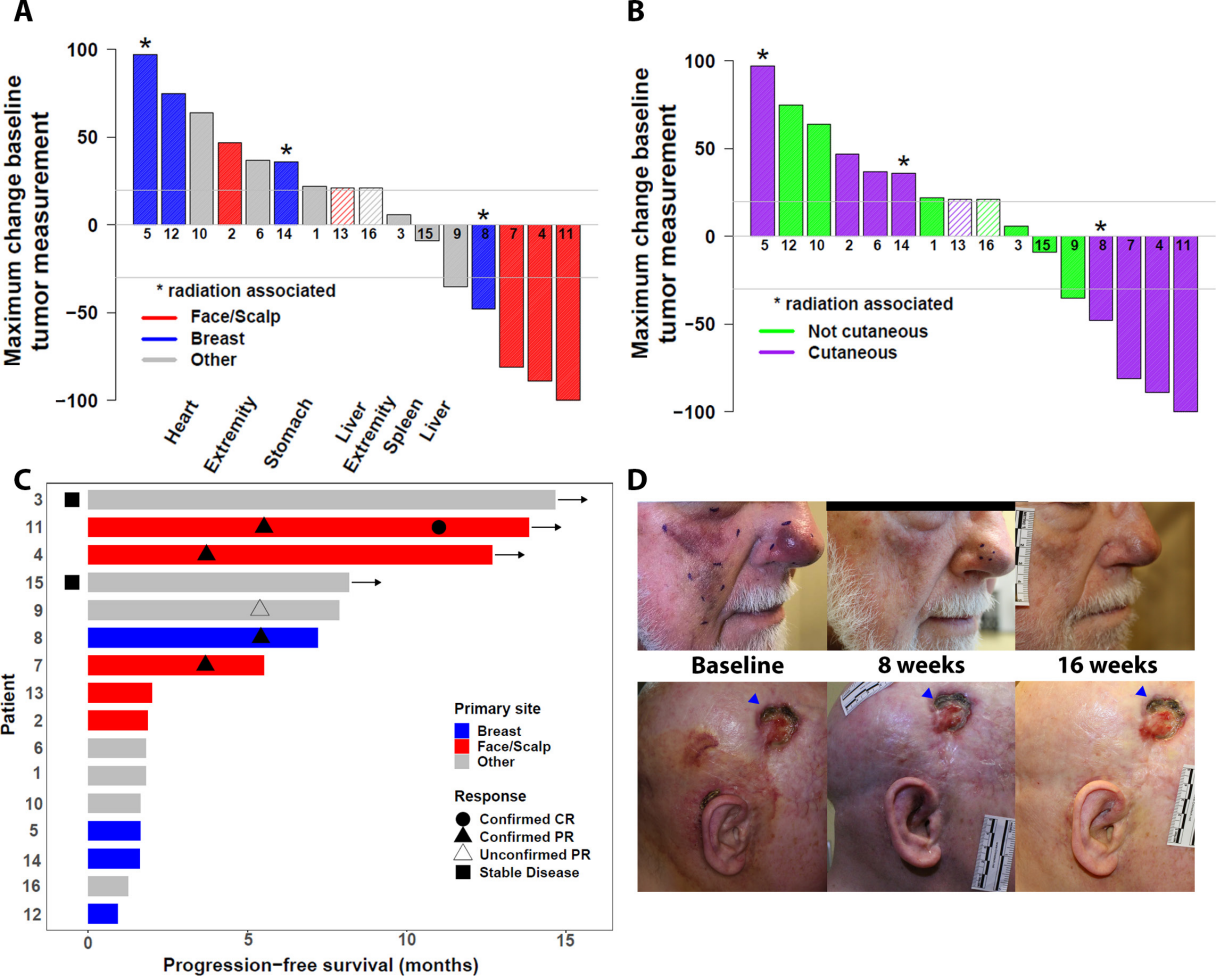
Outcome of patients with angiosarcoma treated with nivolumab and ipilumumab. (A) RECIST waterfall plot by primary anatomic site (patients not assessable by RECIST marked with hatched bars; RECIST progression (+20%) and PR (−30%) marked with horizontal lines); (B) waterfall plot by cutaneous versus non-cutaneous primary site (patients not assessable by RECIST marked with hatched bars; RECIST progression (+20%) and PR (−30%) marked with horizontal lines); (C) Swimmer’s Plot by primary anatomic site; (D) examples of responses, pictures taken at baseline, 8 weeks, and 16 weeks for both patients. Top part of the figure shows a man in his 80s with cutaneous angiosarcoma of the face and one prior therapy who achieved best response of 89% reduction that has lasted 11+ months and is ongoing in spite of a treatment hold for grade 3 elevation of liver transaminases. Molecular alterations showed an intermediate tumor mutation burden (TMB) 8 mut/mb and PDL1 tumor proportion score of 30% (see also table 2); bottom figure is a woman in her 40s with cutaneous angiosarcoma of the scalp and two prior systemic therapies and prior radiation for treatment who achieved best response of 81% tumor reduction that lasted 5 months. There was grade 3 pneumothorax and hypokalemia. Molecular alterations showed a high TMB of 24 mut/mb (see also table 2). Blue triangle points to a chronic lesion resulting from her prior treatments and is not angiosarcoma. CR, complete response; PR, partial response.

**Figure 2:**
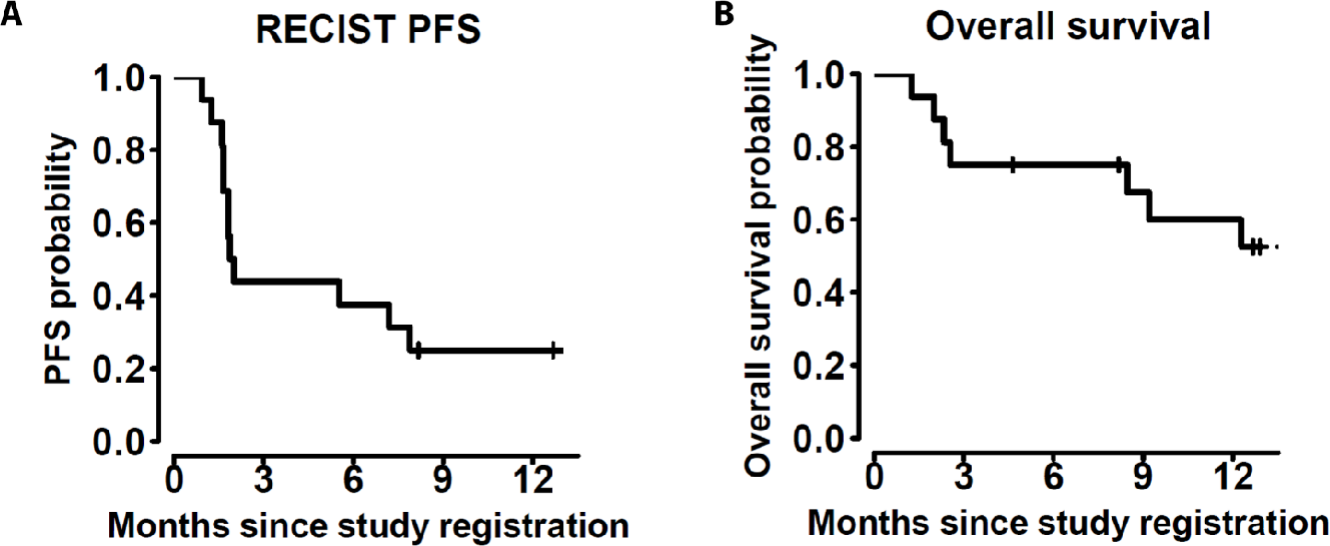
RECIST 1.1 PFS (A) and OS (B) Kaplan-Meier curves. OS, overall survival; PFS, progression-free survival.

Of the 16 eligible patients (online supplemental figure 1), 14 patients were assessable by RECIST; 2 patients were not assessed because they did not have tumor measurements available, having stopped protocol therapy due to AEs before first scan (n=1) or death before first assessment (n=1); these two patients are counted as failures in the ORR calculation. ORR for all 16 patients was 25% (N=4 patients with confirmed response, table 4, figure 1; 95% CI 9% to 45%). These responses have lasted 5, 7, 12+, and 13+ months with the last two responses ongoing. A fifth patient had reduction in tumor size but progression on the follow-up confirmatory assessment. Two patients had stable disease >6 months (6+ and 13+ months). Subgroup analysis revealed that 60% (n=3/5) of patients with primary cutaneous tumors of the scalp or face had a confirmed objective response. Examples of these responses are shown in figure 1D. The fourth objective response occurred in a patient with breast angiosarcoma/post radiation therapy in the study. The overall 6 month PFS rate was 38% (95% CI 20% to 71%, figure 2). The median survival has not been reached after a median follow-up of 12.1 months for patients still alive (figure 2).

10.1136/jitc-2021-002990.supp1 Supplementary data

**Table 4:** RECIST best response summary

	Best RECIST response
Confirmed CR	1 (6)
Confirmed PR	3 (19)
Unconfirmed PR	1 (6)
Clinical benefit (stable disease for 6 months+)	2 (12)
Progression	7 (44)
Not assessed*	2 (12)

N (%) reported.

*Two patients did not have tumor measurements available and were not assessable due to stopping protocol therapy due to adverse events before first scan (n=1) or death before first assessment time point (n=1).

CR, complete response; PR, partial response.

Of the patients for whom molecular information was available, there were two patients who achieved a PR who had data on TMB and PDL1 expression: one of these patients had a TMB 24 mut/mb, and one had TMB 8 mut/mb and positive PDL1 expression (30% TPS) (table 2).

## Discussion

Angiosarcoma has a poor prognosis and improved treatment strategies are urgently needed. Molecular characterization of angiosarcoma has yielded insight into the pathologic drivers of these rare endothelial tumors and the identification of clear subsets of disease. Secondary angiosarcomas were differentiated molecularly from de novo tumors by the presence of *MYC* amplifications^23^; consistent with the literature, our patient with angiosarcoma post radiation to the breast showed a *MYC* amplification as well as, interestingly, an *NTRK1* fusion (table 2). Primary breast angiosarcomas are more likely to harbor *PIK3CA* mutations (as seen in our patient (table 2)).^8^ A subset of angiosarcomas of the scalp and face harbor high TMB and a pattern of DNA damage consistent with ultraviolet (UV) light exposure, suggesting that this subset might be uniquely susceptible to ICI.^8 11^ Indeed, one of the responding patients in our study with angiosarcoma of the scalp had a high TMB (table 2).

Larger studies of immune checkpoint inhibitors in sarcoma have included angiosarcoma patients but in small numbers,^16 24^ making assessment of efficacy in this specific histology impossible from prior studies. Most angiosarcoma responders to immunotherapy in reports where site of primary tumor is available have cutaneous disease of the face or scalp, consistent with the higher TMB seen in this group. At least one response in a radiation-associated angiosarcoma of the breast has also been reported.^19^ There is limited other published data to suggest whether angiosarcomas of other sites might respond to ICI. We, therefore, included all angiosarcomas in this study. To our knowledge, this represents the first prospective trial of immunotherapy in angiosarcoma.

In our cohort, three of the five patients with primary cutaneous disease of the face or scalp had objective responses for an ORR of 60% (figure 1). Surgery and radiotherapy are the current standard of care for localized disease in this specific subset, which necessarily carries high morbidity due to its location. In scalp angiosarcomas, local failure rates in spite of aggressive local treatment are high and long-term survival is poor.^25^ Future studies to incorporate neoadjuvant immunotherapy in this unique population are warranted, as are combination studies to incorporate immunotherapy with frontline chemotherapy. Another response was seen in a patient with a radiation-associated angiosarcoma of the breast, and reduction in tumor size was also seen in a primary tumor of the liver, suggesting that angiosarcoma phenotypes other than that of cutaneous scalp or face tumors also may respond to ICI. Two patients attained stable disease ongoing at 6+ and 13+ months; one had primary angiosarcoma of the spleen and the other had a primary angiosarcoma of the deep soft tissue of the lower extremity. Angiosarcomas that do not have a pattern of DNA mutations consistent with UV light exposure^26^ may have elements of viral DNA,^27^ which may associate with response to ICI in other tumor types.^28–30^

To better understand the molecular basis for response, correlative samples were collected on study and will be analyzed as per the prespecified plan at Cancer Immune Monitoring and Analysis Centers sites. Eight patients had NGS performed for clinical purposes prior to enrollment on the master DART protocol, and results were available as part of the medical record. Consistent with previously published data from other angiosarcoma cohorts, TMB in angiosarcoma was variable. One responder had high TMB (table 2). A second responding patient had strong PDL1 expression on 30% of tumor^31^ and TMB assessed as 8 mut/mb. One patient with a primary visceral angiosarcoma had reduction in tumor size and others had prolonged stable disease, suggesting that these patients may also benefit from ipilimumab and nivolumab. Larger studies allowing for adequate power to stratify subtypes of angiosarcoma are needed to better quantify the potential benefit. This effort will likely require multi-institutional collaboration to enroll sufficient numbers of patients.

Toxicity on this study was comparable to the toxicity seen in other trials with the ipilimumab and nivolumab combination in sarcoma.^16^ The potential clinical benefit of ipilimumab over nivolumab monotherapy in this setting remains uncertain and future trials may better assess the clinical activity versus toxicity profile of nivolumab monotherapy compared with nivolumab and ipilimumab combinations. Similarly, escalating cytotoxic T-lymphocyte associated protein 4 (CTLA-4) inhibition with more frequent dosing or by increasing to a dose of 3 mg/kg as is done in other cancer types, usually with a limited number of ipilimumab doses, may increase efficacy endpoints with the possibility of greater toxicity.^32 33^ A direct comparison would be needed to better quantify alternative dosing schema.

Strengths of this study include the inclusion of patients at both academic and community centers and support from the NCI and SWOG. Since initially conceived, the DART study served an unmet need in that it made an immunotherapy trial available to multiple cohorts of patients with rare tumors, and demonstrated that it was feasible to rapidly accrue even ultrarare tumors.^20^ By adding an angiosarcoma cohort to the DART study, we were able to rapidly accrue this trial for a rare cancer. Weaknesses of the study include a relatively small sample size, precluding our ability to make conclusive comparisons between primary sites or molecular subgroups. Central pathology and radiology review were not mandated; therefore, we relied on local site assessments including from sites that may not have high sarcoma volumes.

Ipilimumab and nivolumab showed activity in angiosarcoma with responses seen in cutaneous angiosarcomas. Correlative studies to better understand the molecular characteristics of these patients utilizing the same centralized platforms are underway. Further study of ipilimumab and nivolumab in angiosarcoma is warranted.

## Data Availability

Data are available on reasonable request. All data relevant to the study are included in the article or uploaded as online supplemental information. Data are currently stored at the centralized SWOG Statistics and Data Management Center, housed at the Fred Hutchinson Cancer Research Center in Seattle, Washington, USA. All relevant data for the angiosarcoma cohort of S1609 are presented in this manuscript. Full protocol and underlying data is available on request.
