# Supplementary material for: Multicenter phase II trial (SWOG S1609, cohort 51) of ipilimumab and nivolumab in metastatic or unresectable angiosarcoma: a substudy of dual anti-CTLA-4 and anti-PD-1 blockade in rare tumors (DART)
Source: J Immunother Cancer. 2021 Aug 2;9(8):e002990. doi: 10.1136/jitc-2021-002990 (PMC8330584; doi:10.1136/jitc-2021-002990)
Supplement: Supplementary data [file jitc-2021-002990supp002.pdf]

Supplemental Table

| Subject | Primary Anatomic Site | Cutaneous vs Not cutaneous | # lines of prior systemic therapy | Best Response | Duration of Response (days) |
|---|---|---|---|---|---|
| 1 | Stomach | Not cutaneous | 1 | PD | |
| 2 | Face/Scalp | Cutaneous | 1 | PD | |
| 3 | Extremity | Not cutaneous | 1 | SD | 446 (ongoing) |
| 4 | Face/Scalp | Cutaneous | 1 | CPR | 386 (ongoing) |
| 5 | Breast | Cutaneous | 1 | PD | |
| 6 | Extremity | Not cutaneous | 5 | PD | |
| 7 | Face/Scalp | Cutaneous | 3 | CPR | 168 |
| 8 | Breast | Cutaneous | 0 | CPR | 219 |
| 9 | Liver | Not cutaneous | 0 | UPR | 240 |
| 10 | Heart | Not cutaneous | 2 | PD | |
| 11 | Face/Scalp | Cutaneous | 2 | CR | 421 (ongoing) |
| 12 | Breast | Not cutaneous | 3 | PD | |
| 13 | Face/Scalp | Cutaneous | 2 | NA | |
| 14 | Breast | Cutaneous | 4 | PD | |
| 15 | Spleen | Not cutaneous | 0 | SD | 249 (ongoing) |
| 16 | Liver | Not cutaneous | 2 | NA | |

PD, progressive disease; SD, Stable disease; CPR, confirmed partial response; UPR, unconfirmed partial response; CR, complete response; NA, not assessed
