# Supplementary figures and images for: Multicenter phase II trial (SWOG S1609, cohort 51) of ipilimumab and nivolumab in metastatic or unresectable angiosarcoma: a substudy of dual anti-CTLA-4 and anti-PD-1 blockade in rare tumors (DART)
Source: J Immunother Cancer. 2021 Aug 2;9(8):e002990. doi: 10.1136/jitc-2021-002990 (PMC8330584; doi:10.1136/jitc-2021-002990)

Supplemental Figure 1: Consort Diagram

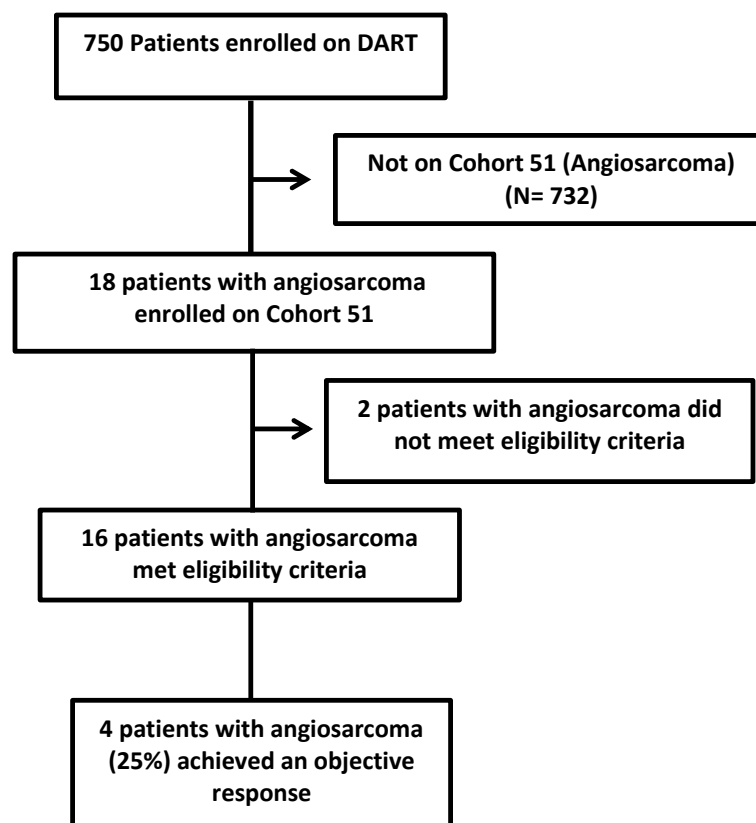

Supplement: Supplementary data [file jitc-2021-002990supp001.pdf]
